# Using the COMMVAC taxonomy to map vaccination communication interventions in Mozambique

**DOI:** 10.1080/16549716.2017.1321313

**Published:** 2017-06-02

**Authors:** Artur Manuel Muloliwa, Julie Cliff, Afiong Oku, Angela Oyo-Ita, Claire Glenton, Heather Ames, Jessica Kaufman, Sophie Hill, Yuri Cartier, Xavier Bosch-Capblanch, Gabriel Rada, Simon Lewin

**Affiliations:** ^a^ DirecçãoProvincial de Saúde, Nampula, Mozambique; ^b^ Faculdade de Medicina, Universidade Eduardo Mondlane, Maputo, Mozambique; ^c^ Community Medicine Department, University of Calabar, Calabar, Nigeria; ^d^ Global Health Unit, Norwegian Institute of Public Health, Oslo, Norway; ^e^ Centre for Health Communication and Participation, School of Psychology and Public Health, La Trobe University, Melbourne, Australia; ^f^ International Union for Health Promotion and Education, Saint-Maurice, France; ^g^ Swiss Tropical and Public Health Institute, Basel, Switzerland; ^h^ University of Basel, Basel, Switzerland; ^i^ Evidence-based Healthcare Program, Pontificia Universidad Católica de Chile, Santiago, Chile; ^j^ Health Systems Research Unit, South African Medical Research Council, Tygerberg, South Africa

**Keywords:** Vaccination, communication, Mozambique, taxonomy, health systems, immunisation, children

## Abstract

**Background**: Improved communication about childhood vaccination is fundamental to increasing vaccine uptake in low-income countries. Mozambique, with 64% of children fully vaccinated, uses a range of communication interventions to promote uptake of childhood immunisation.

**Objectives**: Using a taxonomy developed by the ‘Communicate to Vaccinate’ (COMMVAC) project, the study aims to identify and classify the existing communication interventions for vaccination in Mozambique and to find the gaps.

**Methods**: We used a qualitative research approach to identify the range of communication interventions used in Mozambique. In-depth semi-structured interviews were carried out with key purposively selected personnel at national level and relevant documents were collected and analysed. These data were complemented with observations of communication during routine vaccination and campaigns in Nampula province. We used the COMMVAC taxonomy, which organises vaccination communication intervention according to its intended purpose and the population targeted, to map both routine and campaign interventions.

**Results**: We identified interventions used in campaign and routine vaccination, or in both, fitting five of the seven taxonomy purposes, with informing or educating community members predominating. We did not identify any interventions that aimed to provide support or facilitate decision-making. There were interventions for all main target groups, although fewer for health providers. Overlap occurred: for example, interventions often targeted both parents and community members.

**Conclusions**: We consider that the predominant focus on informing and educating community members is appropriate in the Mozambican context, where there is a high level of illiteracy and poor knowledge of the reasons for vaccination. We recommend increasing interventions for health providers, in particular training them in better communication for vaccination. The taxonomy was useful for identifying gaps, but needs to be more user-friendly if it is to be employed as a tool by health service managers.

## Background

Childhood vaccination is a cost-effective public health intervention that saves millions of lives each year. In recent years, global vaccination coverage for basic vaccines has plateaued at around 86% for three doses of diphtheria–tetanus–pertussis (DTP3) vaccine. In 2014, an estimated 18.7 million infants had still not been reached with routine vaccinations such as DTP3 [1].

Improved communication about childhood vaccination, directed at parents, communities and health care providers, is fundamental to increasing vaccine uptake [2–4]. We define a communication intervention as a purposeful, structured, repeatable and adaptable strategy to inform and influence community decisions in relation to personal and public health participation, disease prevention, health promotion, policy making, service improvement and research [5,6]. While communication is widely seen as important to vaccination programmes, until recently there was no published comprehensive approach to classifying the full range of communication interventions for childhood vaccination. The ‘Communicate to Vaccinate’ (COMMVAC) taxonomy of communication interventions for routine vaccination classifies these interventions into seven categories according to purpose: to inform or educate, remind or recall, teach skills, provide support, facilitate decision-making, enable communication or enhance community ownership. Interventions are also classified according to target groups: (i) parents or soon-to-be parents (ii) communities, community members or volunteers and (iii) health care providers [3]. The COMMVAC taxonomy builds on two broader taxonomies in which the study authors were involved: a comprehensive taxonomy of all interventions for health communication [5] and a taxonomy of interventions that are directed to consumers for evidence-based prescribing [7]. The COMMVAC taxonomy is also informed by the concepts of consumer empowerment and evidence-informed communication and participation, and recognises the multidirectional nature of communication and participation. It is intended to help organise the area of communication interventions for childhood vaccination and to identify the range of communication options that vaccination programme managers can use to improve the uptake of childhood vaccination.

Mozambique, the setting for this study, was estimated in 2016 to have a population of almost 26 million, of whom 68% live in rural areas [8]. Health services are mostly provided by the public sector with coverage estimated at 60% of the population [9]. In addition, there is a large traditional medicine sector and a small private sector [10].

The Expanded Programme on Immunisation (EPI) was introduced in 1979, following a 3 year national vaccination campaign against smallpox, measles, tuberculosis and tetanus [4]. For many years, the routine childhood vaccines included in the EPI were Bacillus Calmette-Guérin (BCG) for tuberculosis, oral polio, DTP and measles, but from 2001 on new vaccines were added: hepatitis B in 2001 [9], haemophilus influenza type B (Hib) in 2008 and pneumococcus (PCV10) in 2011. Planned additions for 2016 are rotavirus, injectable polio and a second dose of measles.

The percentage of children less than 1 year of age who are fully vaccinated has risen from 47% in 1997 to 64% in 2011 [10]. In this period, the Ministry of Health (MoH) has implemented various approaches to increase vaccine coverage. The Reach Every District strategy began in 2008, and aimed to increase services in areas with low coverage, using various operational interventions [11]. National Health Weeks [12] also began in 2008 and are currently scheduled twice yearly. During these weeks, immunisation activities, including information, education and communication (IEC) and social mobilisation, are intensified [13]. Hundreds of mobile teams visit remote communities and schools to vaccinate children, women of childbearing age and primary school students with routine vaccines. Donors provide additional funding for campaigns, while the private sector gives fuel and transport to support vaccination activities and social mobilisation.

National Health Weeks have replaced the campaigns which were carried out in 1998–99 and 2005, with the purpose of controlling or eliminating specific diseases such as measles and poliomyelitis [14,15]. This shift is in line with international policy, which has moved from specific disease campaigns to Periodic Intensification of Routine Immunisation [16]. While the focus of the National Health Weeks is on routine vaccines, their organisation and implementation shares many features with campaigns and this is how we will define them in this paper. The MoH also intensifies communication when new vaccines are introduced, through the media and involvement of government and community leaders at all levels [17].

The objectives of this study were to identify and describe the communication interventions used in both routine immunisation and campaigns in Mozambique and to classify them using the COMMVAC taxonomy. We thus aimed to test whether the taxonomy could be applied to classify communication in campaigns as well as routine vaccination in a low-income country context, and to assess its usefulness in organising and identifying gaps in communication interventions. This research forms part of the COMMVAC project (www.commvac.org), which aims to build evidence for improving communication about childhood vaccination in low and middle income countries. A key part of the COMMVAC project, which ran from 2010 to 2017, was to develop and test a novel taxonomy of vaccination communication interventions for childhood vaccination. Parallel studies to test the COMMVAC taxonomy were carried out by members of the COMMVAC team in Nigeria and Cameroon [18,19].

## Methods

### Study setting and sites

The study was carried out in Mozambique at both the national level and in Nampula Province. The MoH of Mozambique (Ministério da Saúde or MISAU) is organised in three levels: national, provincial and district. At the national level, the MoH has six national directorates, each of which include a number of departments and sections or programmes. Both the EPI and the Health Promotion Department, which collaborates with the EPI to promote childhood vaccination uptake in Mozambique, are located in the National Public Health Directorate. Communication activities for childhood vaccination are supported by the government and by multiple development partners.

Nampula Province, one of Mozambique’s 11 provinces, was purposively selected for this study because the Principal Investigator (PI) is research coordinator at the Provincial Health Directorate and works closely with the vaccination programme. This facilitated the implementation of the study. The province had an estimated population of around five million inhabitants in 2016, corresponding to 19% of Mozambique’s total population [8]. An estimated 77% of the population live in rural areas and 4% are children less than 1 year old. The province is divided into 23 districts, which are further subdivided into administrative posts, localities and communities (rural areas) or neighbourhoods (urban areas). Coverage rates for childhood vaccination in Nampula Province are similar to other provinces in Mozambique with large rural populations.

### Study design

We used a qualitative research approach to identify the range of communication interventions used for childhood vaccinations in Mozambique.

### Data collection

The process of collecting data had several stages, beginning at the national level and continuing in the province of Nampula. Data were collected between February and July 2014. We carried out in-depth and semi-structured interviews with purposively selected key personnel involved in making policy for vaccination and vaccination communication and involved in vaccination delivery at the national level and in Nampula Province (Table 1). In the MoH, we interviewed the EPI manager, EPI communications officer and the deputy head of the Traditional Medicine Department. Within multilateral organisations, we interviewed the United Nations Population Fund, United Nations Children’s Fund (UNICEF) communications officer and the World Health Organization (WHO) logistics officer.

**Table 1.** Overview of study participants.

Level	Interviewees (data collection method)	Number of interviews /group discussions
National	Ministry of Health (interview)	3
	UNICEF (interview)	1
	WHO (interview)	1
Province	Provincial Directorate of Health, Nampula, Mozambique (interview)	2
District	District Community Health Managers (9 participants) (focus group)	1
Health Facilities	Health workers (interview)	8
	Observations (2 urban health facilities and 2 rural health facilities, each of which were observed for one week)	4
Community	Mothers (interview)	8
	Fathers (interview)	8
	Community leaders (interview)	5
	Community Health Workers (interview)	2
	Women (focus groups of 6–12 participants)	3
	Men (focus groups of 10–12 participants)	3

The interviews and group discussions were recorded and transcribed. We also collected documents related to vaccine communication and published since 2000, including reports of activities (*n* = 6), programme evaluations (*n* = 1), reports of study results (n = 1), communication manuals (routine and campaign) (*n* = 5) and strategic plans and strategies (*n* = 3) (Table 2).

**Table 2.** Documents consulted.

Type of document	Document details (translated into English where relevant)
Reports of activities	MISAU*, Semana Nacional de Saúde 1ª fase, 2011 (MISAU, National Health Week 1st phase, 2011).
	MISAU, Semana Nacional de Saúde 2ª fase, 2011 (MISAU, National Health Week 2nd phase, 2011).
	MISAU, Semana Nacional de Saúde 1ª fase, 2012 (MISAU, National Health Week 1st phase, 2012).
	MISAU, Semana Nacional de Saúde 1ª fase, 2013 (MISAU, National Health Week 1st phase, 2013).
	MISAU, Semana Nacional de Saúde 2ª fase, 2013 (MISAU, National Health Week 2nd phase, 2013).
	MISAU, Semana Nacional de Saúde 2ª fase, 2014 (MISAU, National Health Week, 2nd phase, 2014).
Programme evaluations	GAVI, Mozambique pcv10 case report, December 2013.
Reports of study results	De Maria F. Has your child been vaccinated? Report of the Knowledge, Attitudes and Practices (KAP) Rapid assessment study on adherence to vaccination and the introduction of new vaccines. MISAU (Mozambique), UNICEF (Mozambique). 2013. Report.
Communication manuals	MISAU. CIPA – Comunicação Interpessoal e Aconselhamento, Manual de Referência. Maputo. 2002 (MISAU. CIPA – Interpersonal Communication and Counselling, Reference Manual. Maputo. 2002).
	MISAU. CIPA – Comunicação Interpessoal e Aconselhamento, Manual de Formadores. Maputo. 2002 (MISAU. CIPA – Interpersonal Communication and Counselling, Manual for Trainers. Maputo. 2002).
	MISAU. Mobilização Social, in Manual do Programa Alargado de Vacinação. Maputo. 2008 (MISAU. Social Mobilization. In: Expanded Programme on Immunisation Manual, Maputo. 2008).
	MISAU. Vamos descobrir qual é a Importância das Vacinações. Maputo 2012 (MISAU. Let’s Discover the Importance of Vaccinations. Maputo 2012).
	MISAU. Cartão de Saúde da Criança. Maputo. 2010 (MISAU. Child Health Card. Maputo. 2010).
Strategic plans and strategies	MISAU. Mozambique Comprehensive Multi-year Plan (CMYP), 2012 – 2016. Maputo. 2011
	MISAU. Estratégia Nacional para a Promoção da Saúde. Maputo. 2010 (MISAU. National Strategy for Health Promotion. Maputo. 2010).
	MISAU. Plano de Comunicação para a Vacinação de Rotina e Introdução de Novas vacinas 2012–2015: Maputo (MISAU, Communication Plan for Routine Vaccination and Introduction of New Vaccines 2012–2015: Maputo).

*MISAU: Ministry of Health of Mozambique

### Data analysis

A team of two officials in the Health Promotion Department of the MoH worked with the Principal Investigator (AMM) to examine the information obtained from interviews at the national level and from documents, identify communication interventions and classify them according to the COMMVAC taxonomy, which acted as the framework for this analysis. Both officials were experts in communication, and they contributed actively to the classification of interventions.

We then verified the resultant map of communication interventions for completeness through participant observation of routine vaccination in two urban and two rural health facilities in Nampula Province as well as through observation of a campaign. We also interviewed health workers in health facilities and held focus group discussions with District Community Health Managers and parents in communities to identify any further interventions (Table 1). The full COMMVAC study team (all listed authors) later verified and discussed the classifications made using the COMMVAC taxonomy.

### Ethical approval

The proposal was approved by the Unilurio Ethical Review Committee in Mozambique, the ethics review body authorised by the MoH for Nampula Province.

## Results

**Figure 1.**
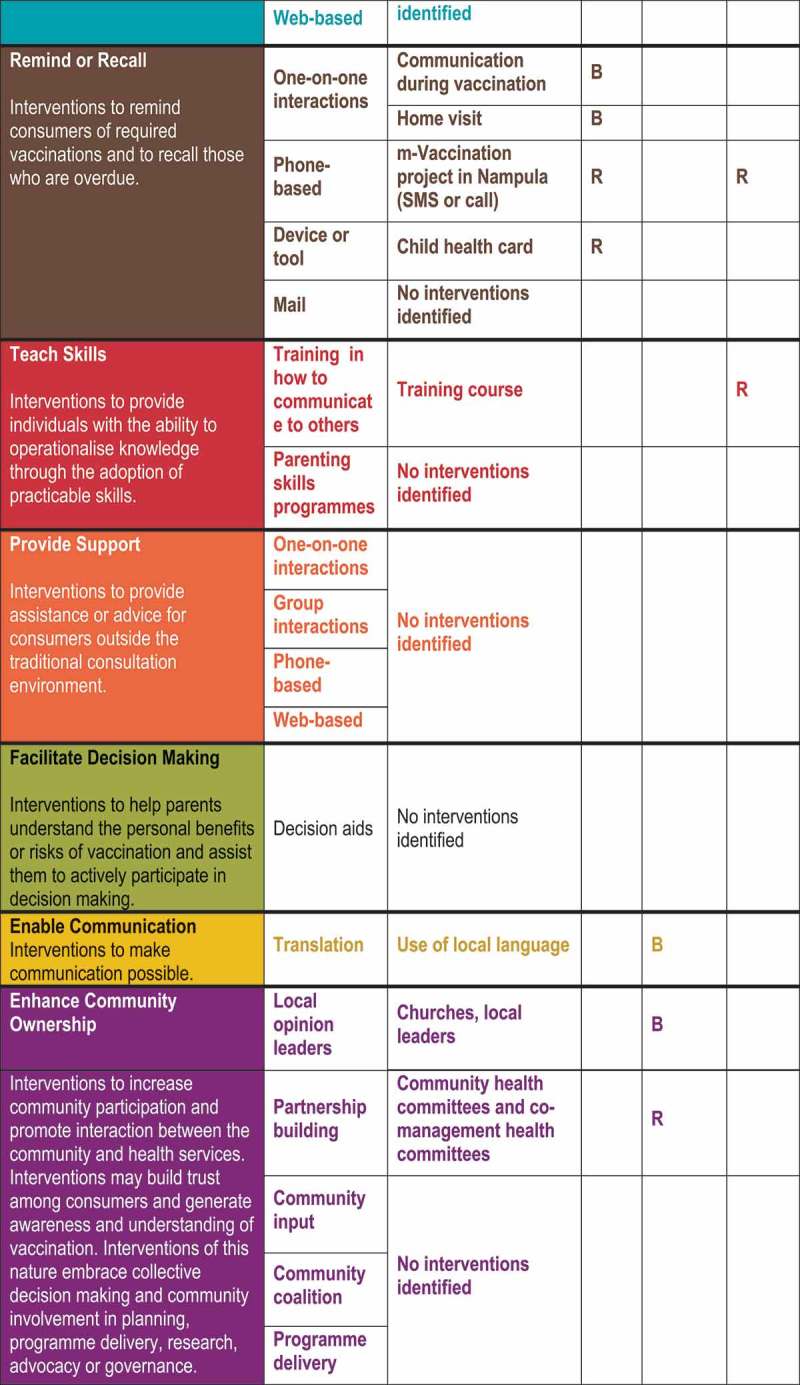
Classification of communication interventions in Mozambique using the COMMVAC taxonomy (Key: C = Campaign, R = Routine, B = Both).

Figure 1 summarises the results of the mapping exercise using the COMMVAC taxonomy. In addition, we specify whether each communication intervention was used in routine vaccination, campaigns or both.

**Figure:**
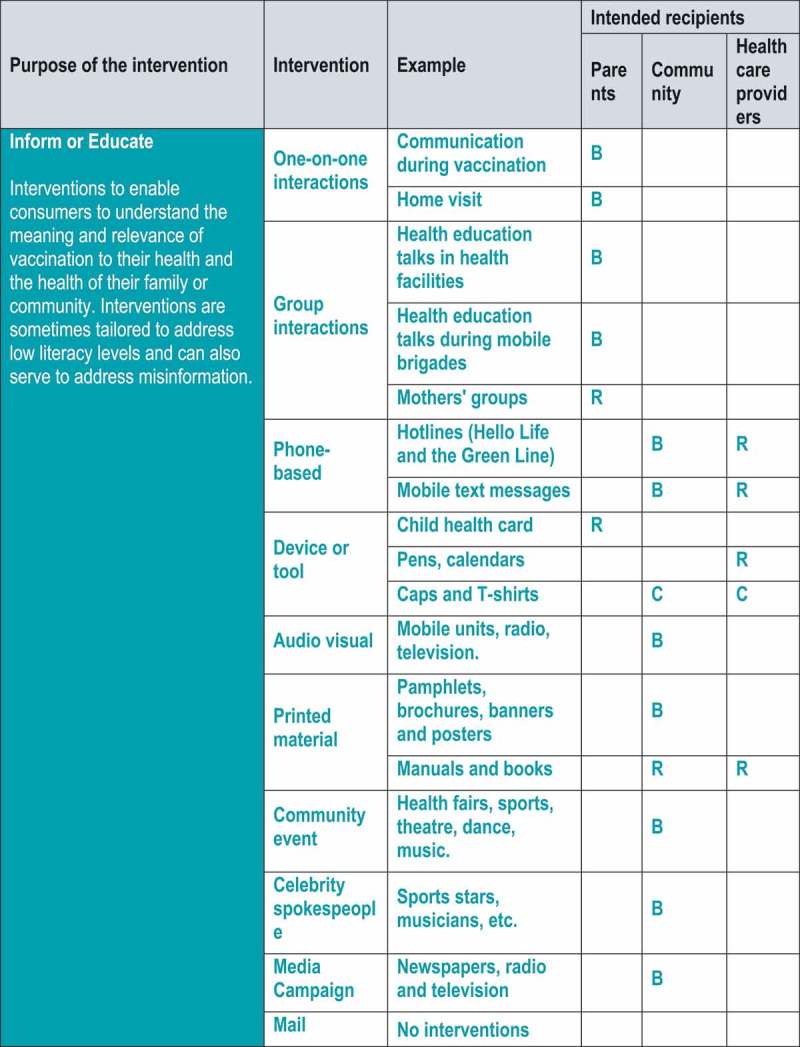

Our identified interventions fitted five of the seven taxonomy purposes. We did not identify any interventions that aimed to provide support or facilitate decision-making. There were interventions for all main target groups, although fewer for health workers. Overlap occurred: for example, interventions often targeted both parents and community members. In the sections below, we discuss in more detail our findings for each intervention purpose.

### Inform or educate

Most of the identified communication interventions had the purpose of informing or educating parents, community members or health care providers (Figure 1). The following types of interventions with this purpose were identified: one-on-one interactions, group interactions, phone-based, device or tool, audio-visual, printed materials, community events, celebrity spokespeople and media campaigns.

#### One-on-one interactions

One-on-one interactions, known in Mozambique as interpersonal communication and counselling, involve direct exchange of information about vaccination between health care providers and parents during vaccination sessions or during a home visit. This offers the opportunity to inform and educate parents during both routine vaccination sessions and campaigns about the names of the vaccines, the diseases they prevent, the vaccination schedule and the importance of meeting it, side effects and their management, and the date that the next vaccination is due. Our observations showed that, in practice, the interaction between the health professional and mothers during vaccination sessions in the health center involved little or no communication about the issues mentioned above.

#### Group interactions

Group interactions are an important part of vaccination communication. The health education talks given to groups of mothers waiting in health facilities or during mobile brigade sessions were the most common group communication intervention associated with both routine vaccination and campaigns. Health facilities have a monthly plan for talks, indicating the presenter and the topic to be covered each day. Vaccination is one of the topics. Other talks cover common diseases, such as malaria and diarrhea. In some areas, mothers’ groups function in the community with the support of non-governmental organisations. The mothers’ groups meet weekly or monthly to discuss health-related themes such as vaccination, infant feeding, pregnancy care and family planning.

#### Phone-based interventions

The MoH has two hotlines available for communication: ‘Hello Life’ and the ‘Green Line’. Hello Life allows the public in any part of the country to call without charge and ask questions on health topics, including vaccination. The Green Line is a free service that allows health workers to ask technical questions on any topic. When a respondent is not available, the caller receives a call back as soon as they are.

The MoH also uses mobile text messaging services to provide information regarding vaccination, mainly during campaigns.

#### Devices and tools

The Child Health Card is a device that contains names of vaccines that each child should receive. The health provider completes this card when the child receives a vaccine. This serves to inform parents of the vaccines their child has received and which vaccinations are outstanding.

Some development partners (UNICEF, United Nations Population Fund and Community Development Foundation) fund sporadic production for health workers of pens and calendars that provide information on vaccination and other health-related topics.

During campaigns, the MoH collaborates with partners to produce T-shirts and caps with vaccination messages, which are distributed to health providers and community leaders. The purpose of these items is to inform communities about the campaign.

#### Audio visual

During campaigns, vehicles equipped with loudspeakers tour the streets in urban centers announcing the dates of vaccination sessions. National, provincial and community radios and television stations broadcast advertisement material and talk shows about vaccination for both routine vaccination and campaigns. Community radios are also used to inform the public of the dates of mobile brigade visits to remote communities.

#### Printed materials

The MoH produces printed materials such as pamphlets, brochures, banners and posters to inform and educate the community about vaccination (Supplementary figures 1 and 2). This material is produced mostly for campaigns. The MoH also produces health education manuals about health topics, including vaccination. These manuals are often directed to specific target audiences, for example health personnel, religious leaders, community leaders, and journalists.

#### Community events

Community events that inform or educate about vaccination include health fairs, sporting events, theatre, music and dance shows. These activities are not focused specifically on vaccination, but are part of health promotion in general. The health fairs, which offer disease screening, are organised by health authorities or other institutions such as the Ministry of Education, the police, and academic institutions, and are often used to commemorate important dates.

#### Celebrity spokespeople

Celebrities, such as sports stars and musicians, are often involved in promoting vaccination messages on radio and television during campaigns and routine vaccination. They participate in advertisements and play songs with vaccination messages, and some are designated as ‘health promotion ambassadors’.

#### Media campaigns

Media campaigns are used widely to inform communities about vaccination or for the introduction of new vaccines. The most frequent use of media involved radio and television spots promoting vaccination, as described above. Health personnel also participate in radio and television programmes. These programmes enable the audience to call in or text with questions.

Newspapers are also used, although their circulation is limited to urban areas. Information in the newspapers is mostly in the form of articles, rather than advertisements.

Launches of campaigns and the introduction of new vaccines at national and provincial levels are covered by the media. The Minister or Vice-Minister of Health typically holds a press conference that receives widespread media coverage.

### Remind or recall

This category encompasses interventions that are intended to remind consumers of required vaccinations and to recall those who are overdue. We describe these interventions below, according to whether they involved one-on-one interactions, were phone based or used a device or tool.

#### One-on-one interactions

Interpersonal communication during a vaccination session and home visits by health care providers or community health workers (CHW) are used to remind mothers of the next date that the child is due for vaccination. Home visits are used more in the context of campaigns than in routine vaccination. During a home visit, the health worker typically reminds the carer of the vaccination campaign dates, checks the Child Health Card for the date of the next vaccination, and reminds parents to take their child for routine vaccination. In 2010, the MoH revitalised its CHW programme. Remote communities choose a CHW and the government provides a six-month training and salary. One of their tasks is to work in the local community to mobilise for vaccination through home visits.

#### Phone-based

In 2014, a mobile phone-based vaccination project was introduced on an experimental basis in Nampula Province. The project uses a mobile phone platform to register information on each vaccinated child, including name, date of birth, place of residence, vaccines received and the phone number of a responsible person. If the child misses the next due date for vaccination, the health provider sends a message or rings to remind parents to take the child to the health facility for vaccination.

#### Device or tool

For routine vaccination, the Child Health Card is a tool that contains information on vaccination and enables health care providers to register the date of the next vaccination. Parents can use this information to recall the date on which they should take the child to the health center.

### Teach skills

This COMMVAC taxonomy category includes all interventions with the purpose of providing individuals with the ability to operationalise knowledge through the adoption of practicable skills. While this category includes both training in how to communicate with others and parenting skills programmes, we did not identify any relevant parenting skills programmes in the study context.

#### Training in how to communicate with others

Health services providers, including vaccinators, receive training in interpersonal communication. The EPI in collaboration with UNICEF delivers this training in a two-day course covering the following topics: use of gestures and words, speaking and listening skills, asking better questions, responding with kindness, and motivating mothers with respect. After the presentation and discussion of each component, participants perform a role-play and the session ends with discussion of the performance. This training has only been offered in some provinces, including Nampula, on a pilot basis, but is due to be scaled up to cover the whole country. The course content lacks specific vaccination information.

### Enable communication

Interventions in this category explicitly and purposefully aim to bridge a communication gap or make communication possible with particular people or groups, as described below.

#### Translation and use of local languages

The translation of vaccination messages into local languages is common in radio programmes. Local languages are also use during health education talks and in one-to-one interactions with parents. The use of these local languages is essential for communication with people who do not speak Portuguese – a majority in rural areas.

### Enhance community ownership

Interventions that aim to increase community participation and promote interaction between the community and health services fall within this taxonomy category. We describe two sets of such interventions below: the use of local opinion leaders and partnership building.

#### Local opinion leaders

Community leaders, such as religious leaders and local political leaders, mobilise people to join in vaccination activities, especially during campaigns, thereby enhancing community ownership. Although traditional healers play a key role in communities, sometimes referring patients to a health facility, their involvement in vaccine communication is minimal.

#### Partnership building

The MoH has a community involvement strategy that aims to enhance community ownership. This strategy builds partnerships through the establishment of health committees and co-management health committees.

Health committees are composed of community members chosen by the community who participate in decisions on community health interventions, including vaccination. These committees are independent of the health facility, but work closely with it. In addition, co-management health committees are composed of health personnel and community members who work together to plan and implement health activities. Both committees meet regularly (monthly, or every 2–3 months) to discuss problems in the health services. They also support the implementation of health promotion in the community, including vaccination.

## Discussion

Despite Mozambique being a low-income country, it uses a wide range of communication interventions for vaccination, although more in the context of campaigns than routine vaccination. A key challenge in resource constrained settings is ensuring that resources for vaccination communication are used as effectively as possible, which requires decisions to be informed by evidence on the effectiveness of different options as well as by information on the acceptability and feasibility of different communication interventions in different settings and groups [20].

Most of the communication interventions for vaccination used in Mozambique had the purpose of informing or educating, as defined by the COMMVAC taxonomy. These interventions are sometimes tailored to address low literacy levels [3]. This is similar to our findings in two lower middle-income countries (Cameroon and Nigeria) where the taxonomy was also used to classify communication interventions [18,19]; in these settings, most interventions also had the purpose of informing or educating. In Mozambique, information and education addressed to groups and individuals with low literacy is particularly relevant for women in rural areas, where literacy rates are low [21,22]. Through information and education, people can understand the significance and relevance of vaccines [3,18,19] which, in turn, may help to reduce vaccine hesitancy [23,24] and encourage people to contribute actively to the vaccination of their children. A recent study in Mozambique found that mothers’ understanding of how vaccines work is limited [20] and that their decision to take their child for vaccination is not based on clear knowledge of the mechanisms of vaccine protection. This suggests a gap in health literacy in Mozambique that has not yet been adequately addressed, and reflects similar gaps in other settings [25,26]. The study suggested that there is a need to move from simple information about bringing babies for vaccination to more explicit information and education on what vaccines are and how they work.

While interventions to remind or recall were used frequently in Mozambique, the range of intervention modalities utilised was narrow, compared to the wide range of such interventions used in many high income countries, which can include letters, emails, postcards, telephone calls and computerised phone messages [3,27,28]. In Mozambique, the most commonly used reminder is the Child Health Card, which is held by every child vaccinated within the routine system. In addition, a number of new technologies are being evaluated in Mozambique, such as the use of mobile phone messaging to remind parents in Nampula Province. The remind or recall interventions that we identified in Mozambique target mostly parents rather than the community as a whole. In the COMMVAC studies in Cameroon and Nigeria studies [18,19], these interventions were more frequently targeted to communities. Health workers or town announcers in schools, churches, used printed materials such as posters, banners, and leaflets to remind or recall in Nigeria; and the mass media (radio and television) and announcements or mosques were used to remind or recall in both Nigeria and Cameroon. In these two countries, the range and frequency of communication interventions for routine vaccination is much greater than that observed in Mozambique, perhaps due to the extra resources available for polio eradication as both countries were still reporting wild poliovirus cases at the time of the studies [29,30].

Teaching skills in Mozambique focused on training health workers in interpersonal communication. In Cameroon, similar training was given to religious leaders involved in promoting vaccination [18]. The EPI organises the training, but it lacks explicit messages or content on vaccination. Including information about vaccination would be a useful addition to the training.

Translation and interpretation are the main communication interventions that enable communication in the Mozambican settings [31]. Mozambique, like most African countries [19], is multilingual with more than 20 languages spoken; many people do not speak the official language of Portuguese [32]. Health messages are written mostly in Portuguese and must then be translated. The degree to which material is translated may require policy decisions and depends on available resources.

The most common interventions to enhance community ownership in Nigeria and Cameroon were the involvement of community leaders or community health committees in vaccination planning or delivery [18,19]. In Mozambique, these approaches are integrated into the MoH’s community health involvement strategy, with the aim of giving communities the power to decide their health priorities.

In Mozambique, no interventions to provide support or facilitate decision-making were identified, similar to our findings in Cameroon and Nigeria.

Most interventions that we identified were directed to community members or parents, and only a few had health providers as targets. This paucity of interventions directed to health providers may have contributed to the poor communication observed between providers and parents during vaccination sessions, a problem also noted in a recent case study for the introduction of the PCV10 vaccine in Mozambique [33].

Many of the communication interventions were used in both routine vaccination and campaigns. In routine vaccination, health education talks predominated, while in campaigns both the range and intensity of interventions were greater, supported by the additional funds available for campaigns, as we found in both Nigeria and Cameroon [18,19]. This increased communication probably contributes to the high coverage rates observed in campaigns, while the routine vaccination programme continues to produce insufficient coverage. This may, in turn, contribute to disease outbreaks and the high morbidity from vaccine preventable diseases observed in some parts of Mozambique [15].

### Strengths and limitations of the study

This study was limited to public sector health services, and did not include the private and NGO sectors. As the public sector is the main entity responsible for vaccination, it is likely that the study identified most of the interventions that are used widely. In addition, we used a range of different approaches to identify communication interventions, including interviews with key national stakeholders and health workers at facility level; group discussions with district managers and parents; observation of routine vaccination in both rural and urban health facilities; observation of campaigns; and document review. We also worked with officials from the Health Promotion Department of the MoH to identify and classify interventions. This enabled us to fulfil the study aims of testing the COMMVAC classification system and identifying gaps in communication interventions. However, we cannot rule out the possibility that not including NGOs or the private sector in the study, and focusing on observing vaccination communication in one province only, may have resulted in an incomplete listing of interventions.

## Conclusions

The communication interventions for vaccination identified in this study mostly served the purpose of informing or educating community members. We consider this focus appropriate in the Mozambican context, where there is a high level of illiteracy and poor knowledge of the reasons for vaccinating children. Overall, we found few interventions directed to health care providers to improve their communication awareness, knowledge or skills. Programme managers should consider increasing the range of interventions directed to this group, and in particular training them in ways of communicating more effectively around childhood vaccination issues.

The COMMVAC taxonomy provided a useful framework for classifying the vaccination communication interventions that we found, and for identifying gaps that may help programme managers identify purposes and target groups that might be missing and decide if their inclusion is warranted. To plan future communication strategies, the taxonomy should be used together with information about the effectiveness of communication interventions for childhood vaccination.

A revised comprehensive taxonomy building on the feedback provided from this and the other field work studies has been developed [34] and work is underway within the COMMVAC project to produce accessible guidance on how the taxonomy can be used by EPI managers and other involved in planning vaccination communication.

## Supplementary Material

Supplementary Material

## Paper context

The COMMVAC project aims to build evidence for improving communication about childhood vaccination. The project has developed a taxonomy of communication interventions as a classification framework. Studies in Mozambique, Nigeria and Cameroon have tested the applicability of the taxonomy in low and middle income countries. In this paper we describe the application of the taxonomy in Mozambique.

## Supplemental material

The supplemental material for this article can be accessed here.
