# Supplementary material for: Using the COMMVAC taxonomy to map vaccination communication interventions in Mozambique
Source: Glob Health Action. 2017 Jun 2;10(1):1321313. doi: 10.1080/16549716.2017.1321313 (PMC5496065; doi:10.1080/16549716.2017.1321313)

**Supplementary figure 1:**


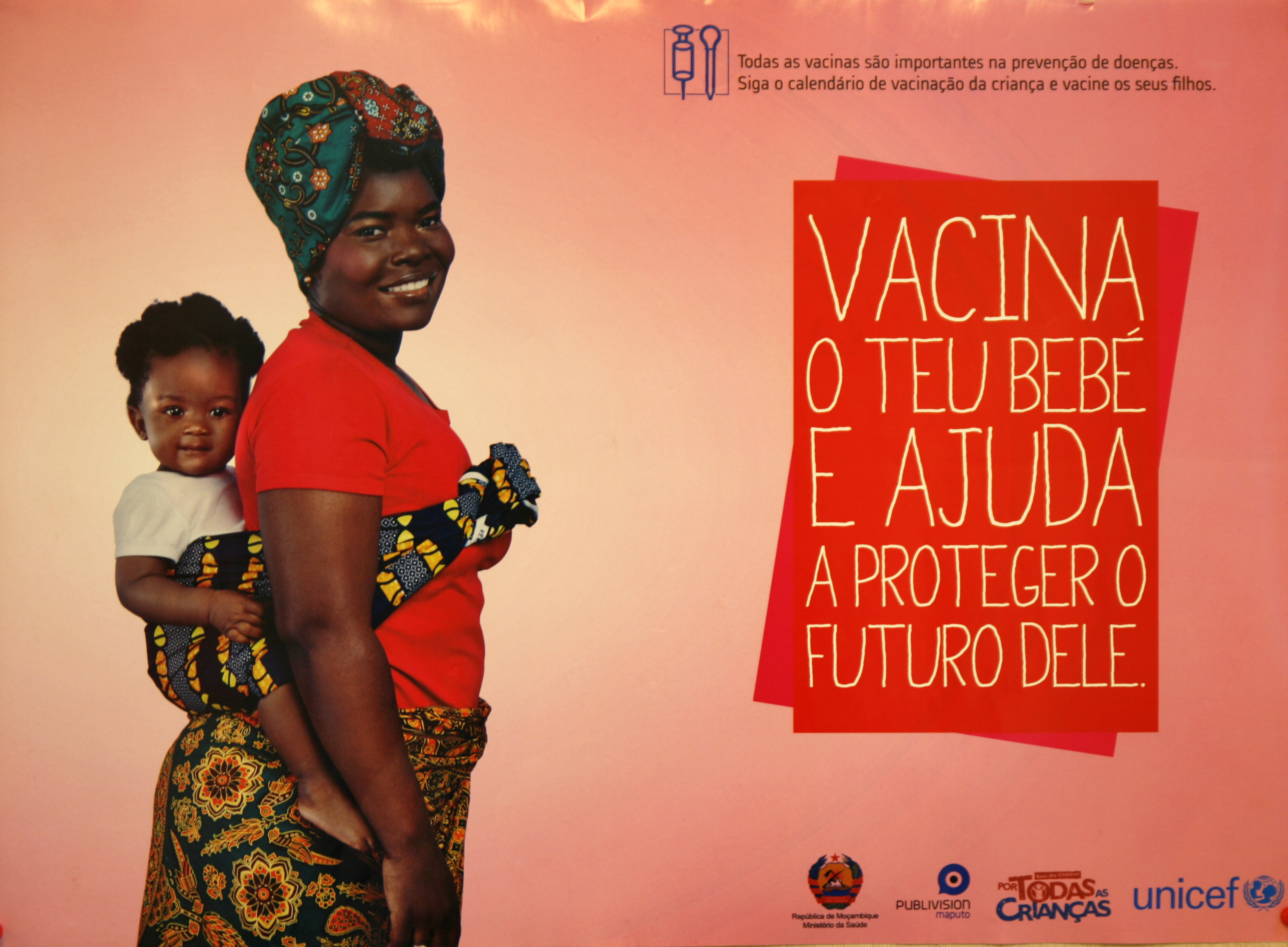


**Supplementary figure 2:**

INCLUDEPICTURE "http://3.bp.blogspot.com/-FEZ2g9IlRyY/VVSbDXIYwUI/AAAAAAAAALc/rWLv9LKNh78/s1600/PARCEIROS.PNG" \* MERGEFORMATINET
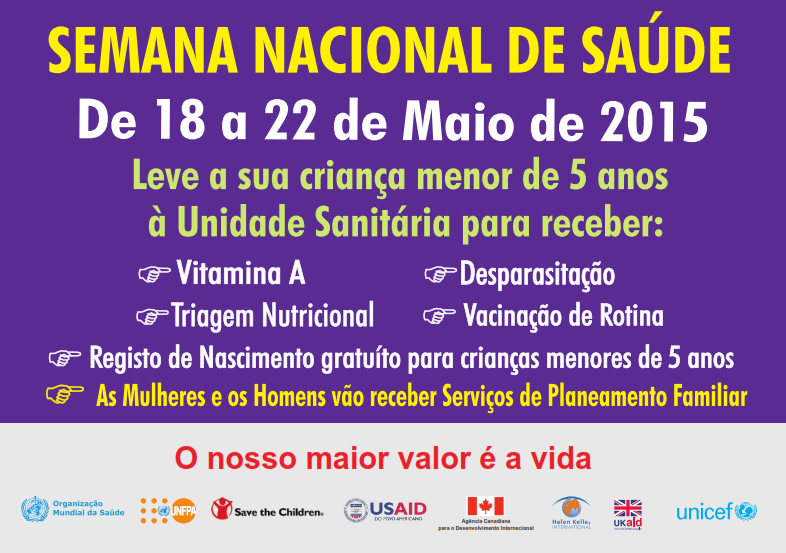

Supplement: Supplementary Material [file ZGHA_A_1321313_SM2539.doc]
